# Development of Immunochromatographic Assay for Determination of Tetracycline in Human Serum

**DOI:** 10.3390/antibiotics7040099

**Published:** 2018-11-13

**Authors:** Anna N. Berlina, Anastasia V. Bartosh, Anatoly V. Zherdev, Chuanlai Xu, Boris B. Dzantiev

**Affiliations:** 1 A.N. Bach Institute of Biochemistry, Research Center of Biotechnology of the Russian Academy of Sciences, Moscow 119071, Russian; berlina.anna@gmail.com (A.N.B.); bartoshasya@yandex.ru (A.V.B.); zherdev@inbi.ras.ru (A.V.Z.); 2 School of Food Science and Technology, Jiangnan University, Wuxi 214122, China; xcl@jiangnan.edu.cn; XCL688@163.com

**Keywords:** tetracycline, human serum, lateral flow, antibiotics detection, immunoassay, gold nanoparticles

## Abstract

Determining antibiotic concentration in human blood provides useful pharmacokinetic information. Commonly used methods such as ELISA require a long time to obtain results and thus cannot be applied when information is needed immediately. In this study, a novel antibody-based lateral flow technique was developed for tetracycline detection in human serum. Contrary to tests developed to analyze food samples, the features of work with serum as analyzed probe were studied for the first time here. The application of labeled and unlabeled specific antibodies was compared. For this purpose, specific and anti-species antibodies were labeled with gold nanoparticles and used for antigen–antibody interaction on the membrane surface with observed staining in the test zone. For both schemes, optimal conditions were established to provide the best sensitivity. The developed assay has a limit of visual detection as low as 35 and 11 ng/mL for the direct and indirect labeled antibodies, respectively. The limit of instrumental detection is from 0.4 to 3.5 ng/mL for diluted and undiluted sera. The use of indirect antibody labeling showed a small increase in sensitivity compared to traditional direct antibody labeling. The developed method showed no cross-reactivity with antibiotics of other classes. The method was used to test samples of serum. The results showed high correlation with the data obtained by ELISA (*R*^2^ = 0.98968). The assay provides a quick assessment of the amount of antibiotics in the blood and keeps them under control throughout the duration of therapy.

## 1. Introduction

Tetracyclines are widely used antibiotics for treating diseases caused by sensitive microorganisms and operate by blocking bacterial protein synthesis, thus creating a bacteriostatic effect on bacterial cells. Introducing tetracycline antibiotics to organisms in various ways leads to their distribution throughout the tissues and biological fluids. Pharmacokinetic investigations using spectroscopic and molecular modeling methods showed that tetracycline binds to site II (subdomain IIIA) of human serum albumin mainly through electrostatic interaction [1]. The main positively charged amino acids involved in this interaction are residues Arg 410 and Lys 414 [1]. Understanding the dynamic of distribution of antibiotics in mammals and humans helps control the treatment process and correct dosages in a timely manner [2]. With the development of antibiotic therapy, bacterial resistance necessitates more careful control of the treatment process. 

The levels of antibiotics depend on several factors. An investigation by Gordon et al. [3] on tetracycline distribution showed that the regime of dosing also matters. After introducing single doses of 250 and 500 mg to health volunteers, tetracycline concentration was determined per hour over 24 h. It was shown that maximum concentration was reached after 3.5–7 h and was in the range of 1.0–2.6 µg/mL after a single dose [3]. Levels of tetracycline in gingival fluid were 2–10 times higher compared to blood. Ahmad et al. [4] showed that variations in dosage protocol influence the level of resistance of bacteria in animal intestines. A toxic concentration of tetracycline in human blood is 30 mg/L, as discovered in an investigation by Schulz et al. [5]. The maximum concentration in human blood was reported as high as 332 ng/mL in an investigation by Liu et al. [6]. A closer, structurally related compound, doxycycline, was observed in nearly the same concentration (3.8 µg/mL) in plasma 2 h after the final dose, in serum at 3.7 µg/mL, and fell to 1.9 µg/mL 12 h after the final dose [7]. Knowledge of these patterns allows doctors to select the most effective antibiotic dosages for therapy. 

To control antibiotic concentration in human serum, various methods are used. Traditional chromatographic methods are used to detect tetracycline in different matrices [8,9]. Methods of liquid chromatography and spectrometry are highly sensitive and precise, but they are expensive and require time-consuming sample pretreatment based on extraction [10,11]. A spectrofluorometric assay requires special conditions and is not widely practiced [12]. As an alternative, immunochemical assays are used in clinical laboratories. A microplate immunoassay with different enzyme labels (EIA) is cheaper but also takes considerable time and laboratory equipment. To make the testing more simple and clear, immunochromatography has advantages including low-cost analysis and the ability to receive quantitative data on a simple and portable optical reader [13,14]. This method has constructive advantages and produces results in a few minutes, making it convenient for mass screening. The principle of the analysis, based on the separation of specific immune complexes from unreacted compounds of human serum, requires minimal sample preparation [15]. 

In contrast to several commercial test systems used to control the amounts of antibiotics in food safety [16,17,18], the tools available to identify antibiotics in serum are limited by immunoenzyme methods. The aim of this investigation is to develop an immunochromatographic assay (ICA) for rapid determination of tetracycline in human serum.

## 2. Results and Discussion

### 2.1. Characterization of Immunoreactants by ELISA

**Figure 1:**
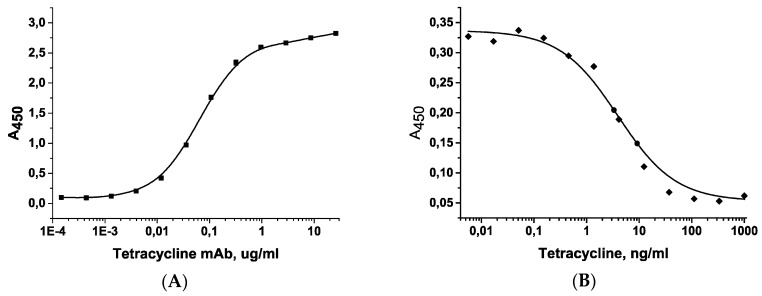
(**A**) Titration of the antibody to tetracycline on immobilized tetracycline-bovine serum albumin conjugate (TET-BSA) conjugate (y = 0.08738 + (2.76344 − 0.08738)/(1 + (x/0.06759)^1.11347^), *R*^2^ = 0.99956); (**B**) calibration curve of competitive determination of tetracycline by ELISA (y = 0.053 + (0.337 − 0.053)/(1 + (x/3.92817)^0.79295^), *R*^2^ = 0.99956).

All reactants such as tetracycline-bovine serum albumin conjugate (TET-BSA) and specific monoclonal antibody (mAb) were initially characterized by ELISA. The binding of antibodies to the detectable hapten, the presence of competition, and primary analytic parameters were evaluated. When antibodies were titrated on the TET-BSA conjugate and immobilized at a concentration of 2 μg/mL, the antibody binding to the hapten was observed (Figure 1A). An antibody concentration of 100 ng/mL was chosen for competitive ELISA, and tetracycline was added to the working buffer (PBST). Figure 1B shows the competitive immune reaction with limit of detection (LOD) of tetracycline 0.2 ng/mL and a working range of 0.7–26 ng/mL.

### 2.2. Choice of Mode for ICA

**Figure 2:**
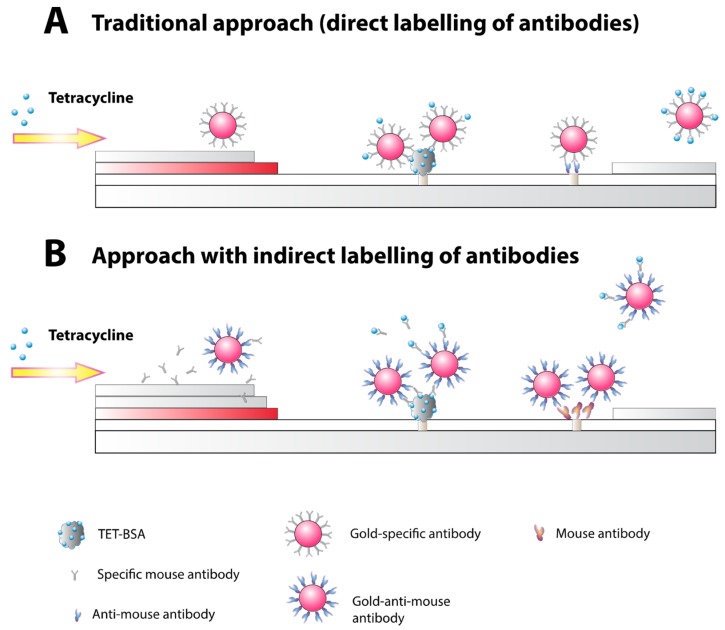
Principle of immunochromatographic determination of TET in human serum: (**A**) with direct immobilization of antibody to the gold nanoparticles; (**B**) with application of native specific antibody and conjugated anti-species antibody.

Due to the low molecular weight of determined analyte, the chosen principle of detection was competitive immunoassay (Figure 2A). To determine the effectiveness of the method for assessing the amount of tetracycline in the serum, two approaches were used. 

Previously, we proposed a new competitive ICA scheme that solves the problem of inefficient binding [19,20]. In the scheme, conjugates of specific antibodies and gold nanoparticles are replaced with a combination of native specific antibodies and anti-species antibodies conjugated to gold nanoparticles. Separating the interaction of the analyzed antigen with specific antibodies and the binding of the formed immune complexes with the nanolabel allows for a significant increase in the ratio (label: specific antibodies) and, therefore, a lower detection limit. At the same time, the high signal (the color intensity in the analytical zone), the reliability of the visual assessment of the assay results, and the accuracy of quantitative determination were maintained. For example, the sensitivity of fumonisin B1 determination increased by 10–20 times [20].

In this study, two approaches were compared to achieve maximum sensitivity: (1) direct immobilization of specific antibodies to gold nanoparticles and (2) the use of native specific antibodies and a conjugate of anti-species antibodies with gold nanoparticles (Figure 2). Both specific and anti-species antibodies were conjugated with gold nanoparticles by physical adsorption [21]. Several conditions were selected: the concentration of the conjugate immobilized on the working membrane, the concentration of the gold conjugate, the number of stages of analysis, and the incubation time. First, TET-BSA and the conjugate of the antibody with gold nanoparticles were immobilized in the corresponding zones on the membranes. Further testing was conducted by immersing the test strips in the tetracycline solution in the working buffer with a low concentration of surfactant Tween 20 (0.05%) or in serum spiked with tetracycline. Preliminary testing in the buffer allowed us to achieve the limit of tetracycline detection at 11 ng/mL in the traditional scheme with direct labeling antibody.

### 2.3. Characterization of the Developed Assay Using Fortified Samples

To analyze blood/serum, samples must be pretreated to avoid the matrix effect. Different approaches are used, such as dilution [22], protein precipitation [23], and extraction [11]. Traditional chromatography methods require the extraction of tetracycline or structurally related compounds by liquid-liquid or solid-phase extraction [7,24].

In this investigation, serum samples were used without sample pretreatment. The series of samples with known spiked concentrations of tetracycline were prepared. ICA was provided in three replicates for each analyte concentration. To decrease the average error of detection and maintain the sensitivity, it is best to use the serum sample as is. Therefore, two variants were tested—undiluted serum and serum diluted 1:1 (*v/v*) with a working buffer. To decrease the viscosity of the serum samples and provide a uniform flow along the working membrane, the surfactant Tween-20 was added at 1% concentration in accordance with our previous studies [15]. Then, the immunochromatographic assay was conducted. 

**Figure 3:**
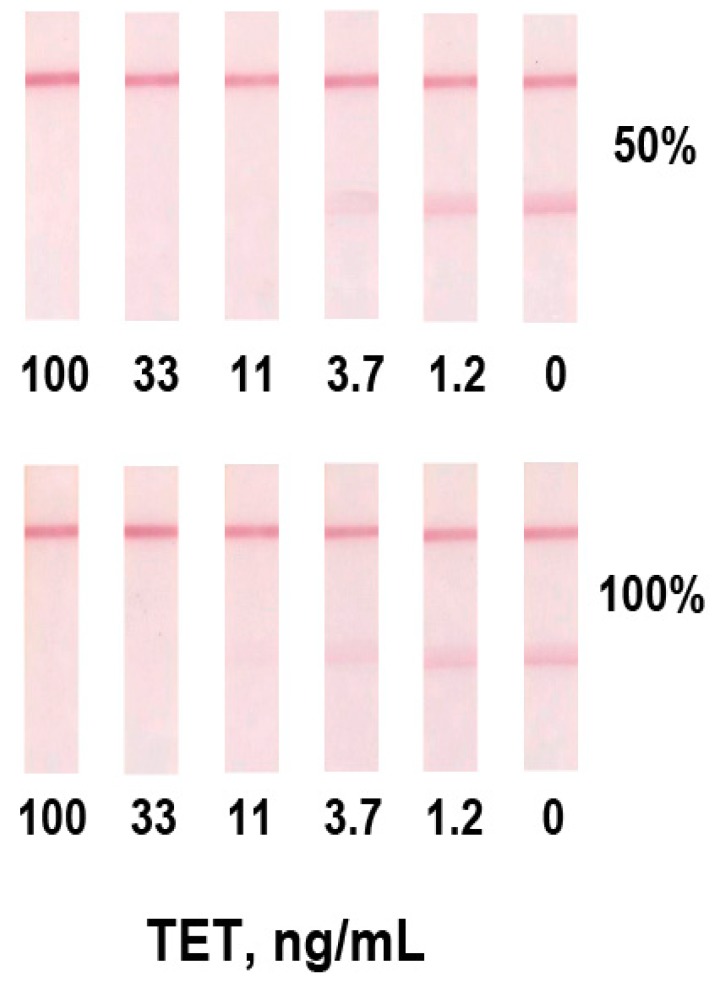
Images of immunochromatographic assay (ICA) test strips with indirect antibody labeling after analysis of serum samples. The strips are arranged according to the tetracycline concentration.

When tetracycline was determined with direct antibody labeling, the limit of visual detection (when test line disappeared) in vitro was 35 ng/mL. At a given analyte concentration, all antibody valencies are occupied and no detectable binding occurs with the hapten-protein conjugate in the test line. Although tetracycline is found in much higher concentrations in blood (at least 1.0 µg/mL [3]) and the achieved sensitivity is sufficient for analytical purposes, we tested the second scheme of the analysis (Figure 2B). In this protocol, a specific antibody at a concentration of 200 ng/mL was used at the competitive stage. Figure 3 shows the absence of a test line at a tetracycline concentration of 11 ng/mL (visual LOD). The obtained data are reproducible both for diluted and undiluted serum. The LOD was the same for both cases.

**Figure 4:**
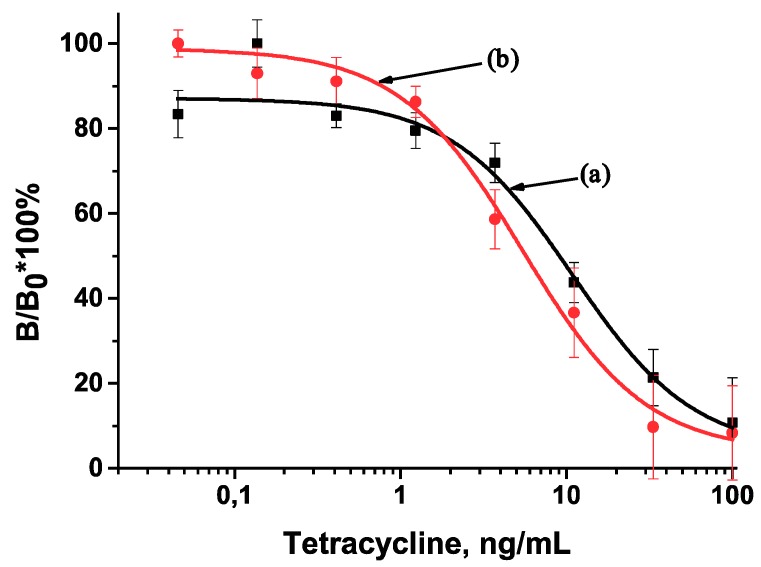
Calibration curves for the tetracycline determination by the developed ICA with direct antibodies labeling: (a) in undiluted serum (y = 4.03194 + (87.06987 − 4.03194)/(1 + (x/10.87484)^1.1857^), *R*^2^ = 0.97758); (b) in twice-diluted serum (y = 3.75211 + (98.79633 − 3.75211)/(1 + (x/5.44732)^1.17032^), *R*^2^ = 0.92389).

**Figure 5:**
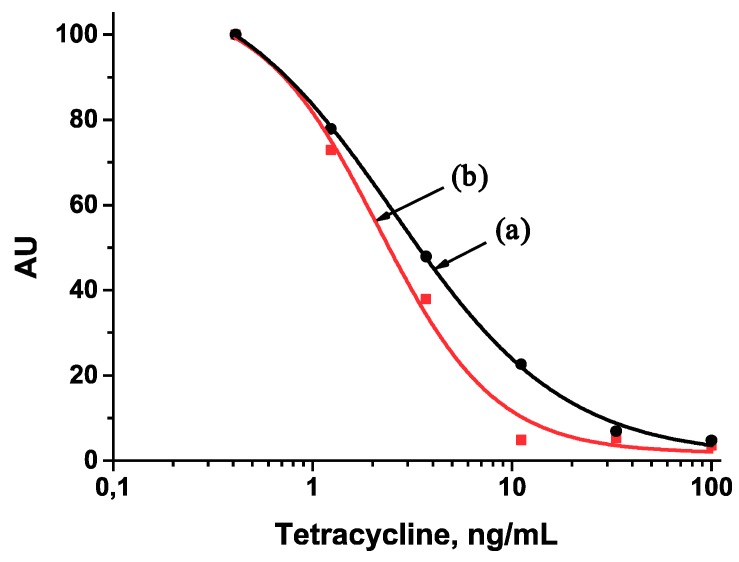
Calibration curves for the tetracycline determination by the developed ICA with indirect antibodies labeling: (a) in undiluted serum (y = 0.8762 + (115.14443 − 0.8762)/(1 + (x/2.57439)^1.01585^), *R*^2^ = 0.9982); (b) in twice-diluted serum (y = 1.74568 + (107.67703 − 1.74568)/(1 + (x/2.13881)^1.47698^), *R*^2^ = 0.98432).

Calibration curves for the TET assay were obtained in diluted and undiluted sera (Figure 4 and Figure 5). Their analytical parameters given in Table 1 indicate advantages of twice-diluted serum and indirect antibodies labeling. Diluting the serum decreases the sample viscosity and provides more mobility to the antibody. While serum dilution does not increase the number of manipulation stages or the ICA duration (10 min), the assay with indirect antibodies labeling demonstrated an increased duration of 25 min. Transferring reactants from solutions to membranes could reduce the ICA duration in the case of indirect antibodies labeling and bring it closer to the ICA with direct antibodies labeling.

The developed assay was checked for selectivity to avoid cross-reactions with antibiotics from other groups widely used for the treatment of bacterial diseases. The results of this evaluation, summarized in Table 2, indicate that the assay is specific to tetracycline and thus provides reliable data about contamination. The obtained data correspond to the results of the previously performed characteristic of the specificity of antibodies used in the work [25], which included seven compounds.

We investigated the recovery of the developed assays. The pooled serum samples were spiked with tetracycline at different concentrations and tested by ICA (added-detected method). The obtained results (Table 3) indicate that both variants of the assay are suitable for detecting tetracycline in human serum.

For 15 serum samples containing tetracycline, parallel testing by ELISA and ICA was performed. The results showed a high degree of correlation, *R*^2^ = 0.98968.

Compared to other methods for tetracycline determination presented in Table 4, our method is competitive for the following reasons. First, the obtained LOD of tetracycline is in accordance with its values in human serum after medical treatment. The second reason is the unique rapidity of immunochromatography, and the third reason is the simplicity of assay implementation. Therefore, the proposed assay is a prospective tool for tetracycline determination in serum during treatment.

## 3. Materials and Methods 

### 3.1. Materials

Mouse monoclonal antibodies against tetracycline (TET) and TET–BSA conjugate were obtained from Jiangnan University (Wuxi, China) [25]. Rabbit anti-mouse polyclonal antibodies were acquired from Imtek (Moscow, Russia). Goat polyclonal antibodies against mouse IgG were procured from Arista Biologicals (Allentown, PA, USA). Anti-species antibody against mouse IgG labelled with horseradish peroxidase were obtained from the N.F. Gamaleya Institute of Epidemiology and Microbiology (Moscow, Russia; www.gamaleya.ru). Albumin from bovines (BSA) was obtained from Boval Biosolutions (Cleburne, TX, USA). Sodium azide, Tween 20, 3,3′,5,5′-tetramethylbenzidine (TMB), and chloroauric acid were acquired from Sigma-Aldrich (St. Louis, MO, USA), and surfactant Triton X-100 was acquired from Panreac Química (Barcelona, Spain). TET was obtained from Applichem (Darmstadt, Germany). All other reagents were of analytical-grade purity or greater. 96-well flat-bottom EIA plates were procured from Costar (NY, USA). The immunochromatographic test strips were fabricated using CNPC 15 type working membrane (mdi Membrane Technologies, Ambala Cantt, India). Deionized water (18 MΩ·cm at 25 °C; Simplicity Millipore, Billerica, MA, USA) was used to prepare all solutions. 

The reagents were applied on the membranes with an IsoFlow dispenser (from Imagene Technology, Hanover, NH, USA). Index Cutter-1 (from A-Point Technologies, Gibbstown, NJ, USA) was used to cut the multimembrane composites into strips. 

Absorbance at 450 nm was measured using Zenyth 3100 microplate reader (Anthos Labtec Instruments, Salzburg, Austria) under ELISA testing of immunoreagents.

Human serum samples from our earlier investigations [15,26] were used in this study and stored at −20 °C.

### 3.2. Antibody Testing by ELISA

TET-BSA was incubated in microplate wells overnight at 4 °C at a concentration of 2 μg/mL in 100 µL of standard ELISA buffer (50 mM phosphate buffer, pH 7.4, PBS). After washing step with PBS with 0.05% Triton X-100 (PBST), 100 µL of a mixture of specific antibodies at a concentration of 200 ng/mL, antigen-containing samples at a concentration of 0.14–100 ng/mL for TET (in PBST) were added and incubated for 60 min at 37 °C. After the incubation, PBST solution of diluted anti-species antibody labelled with peroxidase (1:3000) was added at 100 µL per well and incubated for 60 min at 37 °C. The microplate wells were then washed with PBST. To determine the peroxidase activity, the substrate solution (0.42 mM TMB and 1.8 mM H2O2 in 0.1 M sodium citrate buffer, pH 4.0; 100 µL per well) was added. After incubation at room temperature for 15 min, the reaction was stopped by the addition of 50 µL of 1 M H_2_SO_4_. Then absorbance at 450 nm was measured in each well.

### 3.3. Estimation of the Selectivity of Antibodies Used

For this purpose, tetracycline and three antibiotics (ampicillin, chloramphenicol, streptomycin) from other main groups were chosen as other antimicrobials to check the developed method. Their working solutions were prepared in 20 mM PBS in concentrations ranging from 0 to 1000 ng/mL and tested by ELISA. The cross-reactivity of the antibiotics from other groups was estimated using the followed formula:

CR (%) = (IC_50_TET/IC_50_ antibiotic) × 100

where IC_50_ is the concentration of analyte giving 50% inhibition of the maximum signal detected by ELISA.

### 3.4. Preparation of Gold Nanoparticles and their Conjugation with Antibodies

Gold nanoparticles (GNP; 50 µg/mL concentration, around 30 nm diameter) were obtained according to the Frens’ method [27] with modifications [28] by the reduction of HAuCl_4_ with sodium citrate as a reducing and stabilizing agent. Before the conjugation of GNP and antibodies, the pH of the GNP solution was adjusted to 8.6 with potassium carbonate, followed by the addition of either specific or anti-species antibodies (10 µg/mL of GNP solution) diluted in 10 mM Tris-HCl buffer solution, pH 8.5 (TB) and incubated during 45 min at room conditions. Next, a 10% solution of BSA in Milli-Q water was added (VGNP:VBSA = 40:1), and the mixture was stirred vigorously for 15 min. The resulting conjugate was pelleted by centrifugation at 15,000× *g* for 15 min at 4 °C. The precipitate was resuspended in TB containing 1% BSA, 1% sucrose and 0.05% sodium azide (TBSA). The obtained solution was stored at 4 °C.

### 3.5. Preparation of the Immunochromatographic Test Strips

The TET-BSA conjugate and anti-species (RAMI) antibodies were dissolved in PBS to a concentration of 1 and 0.5 mg/mL, respectively, and then applied on nitrocellulose membranes (CNPC 15 MDI) fixed on a plastic support at a loading of 0.1 µL per 1 mm using an IsoFlow dispenser. The nitrocellulose membrane was connected with the absorbing membrane. 

For strips with direct labeling, antibodies to TET conjugated with GNPs were re-suspended in TBSA with 0.05% Tween 20 with D_520_ = 1.0 and applied to the glass-fiber conjugate pad (3.2 µL per 1 mm). All membranes, working pads, plastic covers, samples, and conjugate pads were collected.

The obtained multimembrane composites were cut into 3.5-mm wide test strips and stored at 20–22 °C in a sealed package containing silica gel.

For strips with indirect labeling, TET-BSA conjugate and mouse antibodies were dissolved in PBS to a concentration of 1 and 0.5 mg/mL and immobilized to the test and control zone, respectively, using the same regime of loading described above. The obtained multimembrane composite was cut the same as for the direct labeling antibody assay. Then, a monoclonal antibody against TET was used as a solution in PBST at a concentration of 200 ng/mL. The conjugate of anti-species antibodies was re-suspended in TBSA containing 0.05% Tween 20 with D_520_ = 1.0 and used as a suspension that was mixed with an antibody solution. 

### 3.6. ICA of TET

The test strips with direct labeling were vertically immersed in two serum samples: (i) 100 µL serum and (ii) mix of 50 µL serum and 50 µL PBST. Surfactant Tween-20 was added to all solutions with 1% concentration. Each test strip was incubated for 5 min. After one minute of drying, the test strip was scanned. The total analysis time was 6–7 min. 

The test strips with indirect labeling were vertically immersed in two versions of a serum sample: (i) mix of 100 µL serum with specific antibody (200 ng/mL) and (ii) mix of 50 µL serum with specific antibody (200 ng/mL) in 50 µL PBST. Surfactant Tween-20 was added to all solutions with 1% concentration. Each test strip was incubated for 5 min. Next, the test strips were incubated for 20 min in a solution of secondary (anti-species) antibodies conjugated with gold nanoparticles (A525 = 1). After one minute of drying, the test strip was scanned. The total analysis time was 26–27 min.

### 3.7. ICA Data Processing

After incubation, the strips were scanned in a flatbed scanner (Canon Lide 90, Canon, Tokyo, Japan, canon.com) with a resolution of 600 dpi without applying modes for contrast or color correction. The intensities of coloration were quantified using the Total Lab software package (TotalLab, Newcastle upon Tyne, UK). 

The dependences of color intensity from the TET concentration in the sample were plotted using Origin 9.0 software (Origin Lab, Northampton, USA, origin lab.com) and approximated using four-parameter sigmoid function y = (A − D)/(1 + [x/C]^B) + D, and the working range for quantitative detection of TET (IC_20_ and IC_80_) was calculated. The visual detection limit for GNP binding in the test zone corresponded to the recorded integrated color intensity equal to 25 arbitrary units (AU). The instrumental detection limit corresponded to IC_10_.

## 4. Conclusions

We have developed the first immunochromatographic assay of tetracycline in human serum that combines rapid analysis and semi-quantitative (visual) or quantitative (instrumental) estimation of results. The assay detected tetracycline in undiluted and diluted sera in a working range of 3.8–35.0 and 1.7–17.8 ng/mL, respectively, using direct antibody labeling. When indirect antibody labeling was used, these parameters were 0.7–10.1 and 0.8–5.4 ng/mL for undiluted and diluted sera, respectively. The obtained high recovery values of 92–132% as well as the high correlation with ELISA results (*R*^2^ = 0.98968) indicate the competitiveness of the assay and its aptitude for tetracycline detection in human serum. 

The developed assay is fast, precise, and sensitive. It is also notable for its simple serum preparation by dilution. Therefore, the assay can be used for mass screening of samples or for monitoring one patient to assess the effectiveness of medication. The assay does not require special staff training and has a relatively low cost. The universality of the realized approaches also allows them to be applied to other antibiotics.

## Figures and Tables

**Table 1:** Analytical parameters of two tetracycline ICA schemes (*n* = 3).

Sample	IC_20_^1^, ng/mL	IC_50_, ng/mL	IC_80_^2^, ng/mL
Direct antibody labeling			
Undiluted serum	3.8 ± 1.4 *	10.9 ± 5.9	35.0 ± 14.4
Serum diluted 1:1 (*v/v*) with buffer	1.7 ± 0.3 *	5.4 ± 1.4	17.8 ± 8.1
Indirect antibody labeling			
Undiluted serum	0.7 ± 0.2	2.6 ± 0.3	10.1 ± 1.2 *
Serum diluted 1:1 (*v/v*) with buffer	0.8 ± 0.1	2.1 ± 0.2	5.4 ± 1.1 *

* statistically significant differences. ^1^ Limit of instrumental detection. ^2^ Limit of visual detection.

**Table 2:** Parameters of specificity in the developed immunochromatographic assay.

No.	Antibiotic	IC_50_, ng/mL	CR, %
1	Tetracycline	8.1	100
2	Ampicillin	>5000	<0.1
3	Chloramphenicol	>5000	<0.1
4	Streptomycin	>5000	<0.1

**Table 3:** Determination of tetracycline in human serum by the developed assay (*n* = 3).

Fortification Level, ng/mL	Recovery ± Standard Deviation (%)	
	Undiluted Serum	Serum Diluted 1:1 (*v/v*)
35	121.4 ± 7.7 *	132.0 ± 12.2 *
12	113.8 ± 9.8	116.7 ± 10.5
4	92.0 ± 4.6 *	108.6 ± 7.9 *

* statistically significant differences.

**Table 4:** Methods of detection tetracycline and structurally related compound doxycycline in human serum.

LOD, Working Range (ng/mL)	Method	Kind of Sample	Specific Conditions	Ref.
332	LC-MS/MS	Human serum	Extraction of tetracycline	[6]
200,400−4000	Spectrofluorometry	Human serum	Addition of Mg^2+^ for tetracycline detection	[12]
50–6000	LC-MS/MS	Human plasma	Protein precipitation for assay of tetracycline and oxytetracycline	[11]
125,250–5000	HPLC	Human plasma, tissues	Solid-phase extraction of doxycycline	[7]
20–1600	LC-MS	Human plasma	Protein precipitation for assay of doxycycline	[23]
100,500–5000	LC-MS	Human plasma	Liquid-liquid extraction of doxycycline	[24]
11 (visual LOD)	ICA	Human serum		This study
